# Towards CMOS Integrated Microfluidics Using Dielectrophoretic Immobilization †

**DOI:** 10.3390/bios9020077

**Published:** 2019-06-05

**Authors:** Honeyeh Matbaechi Ettehad, Rahul Kumar Yadav, Subhajit Guha, Christian Wenger

**Affiliations:** 1 IHP-Leibniz-Institut für innovative Mikroelektronik, Im Technologiepark 25, 15236 Frankfurt/Oder, Germany; yadav@ihp-microelectronics.com (R.K.Y.); guha.ihp2608@gmail.com (S.G.); wenger@ihp-microelectronics.com (C.W.); 2 Brandenburg Medical School Theodor Fontane, 16816 Neuruppin, Germany

**Keywords:** biomolecules, microfluidic, dielectrophoretic immobilization, CMOS biosensor, lab-on-chip

## Abstract

Dielectrophoresis (DEP) is a nondestructive and noninvasive method which is favorable for point-of-care medical diagnostic tests. This technique exhibits prominent relevance in a wide range of medical applications wherein the miniaturized platform for manipulation (immobilization, separation or rotation), and detection of biological particles (cells or molecules) can be conducted. DEP can be performed using advanced planar technologies, such as complementary metal-oxide-semiconductor (CMOS) through interdigitated capacitive biosensors. The dielectrophoretically immobilization of micron and submicron size particles using interdigitated electrode (IDE) arrays is studied by finite element simulations. The CMOS compatible IDEs have been placed into the silicon microfluidic channel. A rigorous study of the DEP force actuation, the IDE’s geometrical structure, and the fluid dynamics are crucial for enabling the complete platform for CMOS integrated microfluidics and detection of micron and submicron-sized particle ranges. The design of the IDEs is performed by robust finite element analyses to avoid time-consuming and costly fabrication processes. To analyze the preliminary microfluidic test vehicle, simulations were first performed with non-biological particles. To produce DEP force, an AC field in the range of 1 to 5 V (peak-to-peak) is applied to the IDE. The impact of the effective external and internal properties, such as actuating DEP frequency and voltage, fluid flow velocity, and IDE’s geometrical parameters are investigated. The IDE based system will be used to immobilize and sense particles simultaneously while flowing through the microfluidic channel. The sensed particles will be detected using the capacitive sensing feature of the biosensor. The sensing and detecting of the particles are not in the scope of this paper and will be described in details elsewhere. However, to provide a complete overview of this system, the working principles of the sensor, the readout detection circuit, and the integration process of the silicon microfluidic channel are briefly discussed.

## 1. Introduction

Dielectrophoresis (DEP) is the change in the dielectric behavior of the particles placed in a non-uniform AC electric field [1]. When particles are subjected to this AC field, dipole moments are induced in the particles. The particle movement is dependent on the polarizability of the particle relative to its suspending medium. The DEP force is strongly dependent on the particle diameters, E-Field vectors, frequencies, and dielectric properties of the particles and their surrounding medium. The Clausius Mosotti (CM) factor depends on the properties of the particle and the medium, as well as the frequency of the exciting field [2]. The permittivity of the particles is the dominating parameter affecting the CM factor. The frequency of the DEP field is unique for every particle. The CM factor defines the sign of the DEP force. Positive CM values lead to a positive DEP (pDEP) force on the particles, pushing them towards the denser E-Field regions. This occurs when the polarizability of the particle is larger than the polarizability of the fluid. Negative CM values result in negative DEP (nDEP) and drive the particles away from the dense E-Field regions [3,4]. DEP techniques are widely used in microfluidic biochips, so-called lab-on-a-chip (LOC) devices [4,5,6,7]. Based on the size and dielectric properties of the particles, DEP can be applied for manipulation of the particles. In this work, positive DEP is used to immobilize particles on the electrodes. By combining the capabilities of complementary metal-oxide-semiconductor (CMOS) based LOC platforms with sensor elements, also called biosensors, the microsystems enable fast diagnostic results [8,9,10,11]. The interdigitated biosensor has been previously used to immobilize proteins [12], polystyrene beads, and biological entities [5] as well as detecting organic fluid’s dielectric constants [13], suspended biological cells [14], and creatinine molecules [15]. Over recent decades, many investigations have been performed on the detection of bioparticles with CMOS integrated microfluidic systems [7,13,16,17,18,19,20,21]. Among these investigations, some of the systems were benefited from the sensing and detection on the same BiCMOS integrated platforms. However, using a single sensor for dielectrophoretic manipulation as well as sensing/detecting accompanied by microelectronics circuitry for readout on one single chip has not yet been explored. Table 1 presents a comparison of the reported techniques using both DEP and without DEP on a CMOS platform for biological applications.

Originally, Otto et al. [7] described the use of DEP as a simple technique to immobilize the protein particles on conductive surfaces of a CMOS device. These surfaces could be small active regions of micrometer size. Moreover, antibodies were immobilized on the conductive surfaces permanently using DEP. Although this device presented the potential for integration with the microelectronics circuitry, the circuits for data acquisition and processing was omitted. Manaresi et al. [16] developed a LOC architecture based on a two-dimensional array of microsites on a CMOS microsystem for manipulation and detection of individual cells on a standard CMOS microsystem. The 8 × 8 mm^2^ chip included 102,400 actuation electrodes, embedded memory for electrode programming, and an optical sensor. Although manipulation and detection of cells occurred in a parallel manner, generating the electric field to create DEP cage and cell detection was obtained through different units. To create a DEP cage, counter electrodes surrounded by in-phase electrodes were used. The optical sensor detected the presence of the trapped cells. Miled et al. [17] proposed a microfluidic structure containing 64 electrodes for the manipulation of cells. These electrodes were capable of moving, separating, and rotating the cells. Additionally, an interdigitated electrode was embedded in the microfluidic channel. This electrode was responsible for detecting the microfluidic channel capacitance alteration and was connected to a CMOS chip for data acquisition. A CMOS chip monitored the DEP dependent variables (e.g., frequency and voltage) to evaluate the cross-over frequencies, which can be characteristic for each cell. However, this system does not benefit from simultaneous cell manipulation and detection. Another CMOS based LOC platform was developed in the work of Park et al. [18] for trapping, rotating, and detecting cells and microorganism using DEP. The proposed system used octa-pola electrodes to trap and rotate the particles. Simultaneously, these particles were detected by in-situ impedance monitoring on the same chip. This single chip platform included an on-chip Trans-Impedance Amplifier wire bonded to a printed circuit board (PCB) with external circuits to readout the change in impedance. To avoid the contact of aqueous biological media with the electronics of the chip, polydimethylsiloxane (PDMS) wells were fabricated on the chip.

Guha et al. [19,20,21,22] has extensively worked on developing single BiCMOS LOC platforms for biological cell sensing and detection using different techniques. In all of these works, the sensing principle of this sensor is based on the relative permittivity change of the material-under-test (MUT). Variation in biological cell resulted in the fringing field capacity change of the sensor, which caused capacitance variations to be detected by an associated silicon high-frequency readout circuit. By applying electric potential to the electrodes, a non-uniform electric field between the adjacent fingers is generated. This method is used to detect the dielectric permittivity of the MUT. Guha et al. [19] presented a CMOS based high-frequency sensor with the capability of distinguishing the blood sample with fat and calcium from the normal blood sample. The sensor is placed at the top and inside the wall of the catheter and exposed to the blood. In other work [20], he proposed the integration of the silicon microfluidic channel with a CMOS sensor circuit for cytometric applications to detect the cells concentrations using dielectric spectroscopy. In this method, the interdigitated electrode (IDE) arrays were placed on top of the microfluidic channel. As a result of IDE excitation, the fringing electric fields penetrate into the fluid flowing through the channel. They also investigated the variation of the relative viscosity in an aqueous solution [21] using radio frequency (RF) CMOS chem-bio sensor. The fringing field between the adjacent fingers is utilized to detect the dielectric permittivity of the MUT. Furthermore, he developed a self-calibrating highly sensitive dynamic IDE sensor in a BiCMOS based PDMS microfluidic platform that can be applied for particle counting and single particle sensing in a fluidic system [22]. The steady flow of the particles suspended in the fluid results in capacitive pulses from the sensor that is embedded in the oscillator. Eventually, these pulses translate to frequency modulation using an integrated phase-locked loop demodulator.

In this work, an IDE sensor is proposed and designed to immobilize the particles using the DEP technique. This IDE platform can be integrated with circuits and microfluidic channel by CMOS process line of IHP-Leibniz-Institut für innovative Mikroelektronik, for simultaneous immobilization, sensing, and detection of particles. The geometry of the IDEs has been adapted from the previous works done at IHP [14,15]. This work aims to enable the design of an IDE with the capability to immobilize micron and nano-sized particles. By placing an AC voltage source to the electrodes, a non-uniform electric field is generated. The gradient of the electric field is maximized at the edges of the IDE structures. Consequently, particles can be immobilized on the electrodes. To optimize the efficiency of the IDEs and DEP force for immobilizing different particle sizes, numerical simulations were done to study the effect of electric potential, frequency, fluid flow, and geometrical parameters of the IDE on particle tracing in a feasible manner. Different geometrical parameters of the IDE, such as finger width and spacing, were modeled. Finite element analysis was performed to evaluate the best parameters for dielectrophoretic immobilization. The first simulation model for the particle tracing was presented previously [23]. The details of the simulation with the extended versions are presented in this work.

## 2. Sensing and System Overview

This section describes the subsets of the advanced technological process required for enabling the dielectrophoretic immobilization of the bio-particles. The details of the IDE prototype used for manipulation and sensing as well as the readout detection circuit design along with the silicon microfluidic channel integration technology are presented below.

### 2.1. Silicon Interdigitated Capacitor Sensor

**Figure 1:**
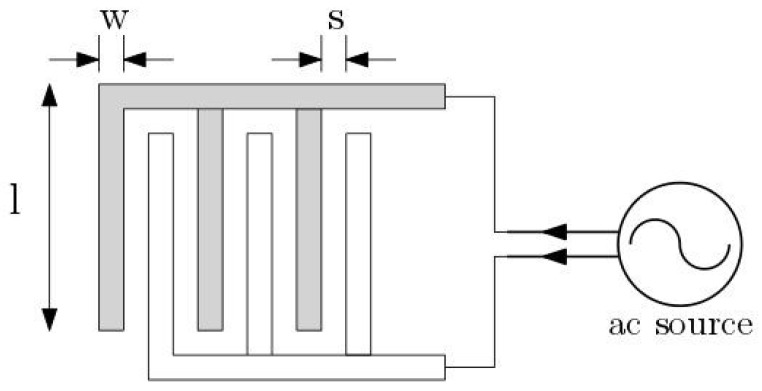
Multi-fingered planar interdigitated electrodes (IDEs).

The IDE used in this work is similar to a sensor previously established for nearfield biosensors [14,15]. The fabrication of the IDE was performed based on the standard 250 nm high-performance SiGe BiCMOS technology of IHP. This sensor unit is embedded in the microfluidic channel and relies on the multi-fingered planar interdigitated capacitor arrays (see Figure 1), which are fabricated on the topmost metal level of the back-end-of-line (BEOL) stack of the CMOS/BiCMOS process. This IDE is used to model the dielectrophoretic structures.

**Figure 2:**
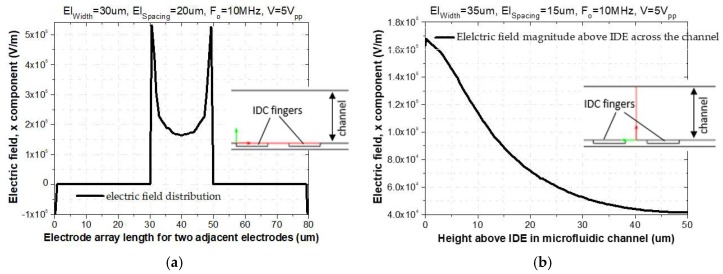
(**a**) E-Field distribution above two adjacent fingers of IDE; (**b**) Electric field magnitude as a function of distance above IDEs.

As shown in Figure 2a, the magnitude of the fringing electric field is enlarged between two adjacent fingers of the IDE. As a result of the increased gradient of the E-Field at rectangular electrode corners, the distribution of the E-Field is inhomogeneous [24]. This effect leads to attraction and trapping of the particles to this region. As shown in Figure 2b, this E-Field magnitude exponentially decays with increasing distance to the IDE arrays. To visualize the simulated electric field, a cut line 2D data set was used to create lines through the modeled 2D geometry, as shown in cross-sectional schematic figures below. The electric field has been plotted along the red arrow-line, Figure 2a,b. The distribution of the electric field is calculated based on Equation (10).

**Figure 3:**
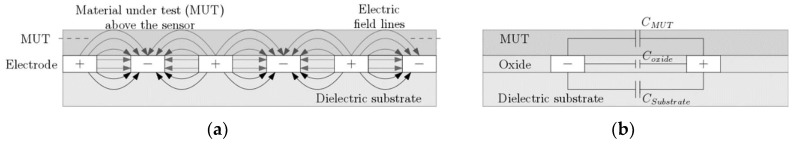
Interdigitated electrode: (**a**) Electric field distribution of the IDE; (**b**) Equivalent capacitive contributions from IDE and material-under-test (MUT).

The sensing principle of the IDE is based on the variation of the fringing electric field between the fingers of the IDE due to the permittivity change of the material (medium), as shown in Figure 3a.

The capacitance of the fringing field can be analyzed based on the quasi-static approximation of Maxwell’s Equation [18]. The total capacitance of the two consecutive electrode fingers is equal to the sum of the contributing capacitors, as shown in Equation (1) [14]. This capacitance results from the penetration of the fringing field into the MUT as well as substrate and influence of the finger height on parallel plate capacitance, Figure 3b.

(1) $$C_{total}=C_{MUT}+C_{Substrate}+C_{Oxide}.$$

The capacitance of the fringing filed concerning MUT and substrate is given as below [17].

(2) $$C_{MUT}+C_{Substrate}=\;\varepsilon_{0}\left(\frac{\varepsilon_{substrate}+\varepsilon_{MUT}}{2}\right)\left(\frac{K\sqrt{1+k^{2}}}{K\left(k\right)}\right),.$$

where *K*(*k*) is the parameter arising due to the conformal mapping technique. The change in total capacitance is governed by the permittivity of the *MUT*, as shown in Equation (2).

The sensing operation of the IDE relies on the electrodynamics of the parallel plate capacitors. Applying an AC voltage to positive and negative terminals, a non-un electric field is created in the vicinity of the IDE. This electric field impacts the trajectory of the particles which are suspended in the medium above the IDE passing through the microfluidic channel. The capacitance of the IDE (sensor) changes, as a result of the permittivity change and its geometry [25]. Thus, the varying value of the IDE’s capacitance alters the impedance of the IDE which can, in turn, be deduced through impedance spectroscopy. One such readout architecture which characterizes a sensor is a reflectometer. It measures the change in reflection amplitude and phase from a sensor (here IDE), which is a function of the change in sensor impedance. This readout scheme is elaborated further in the succeeding sub-section.

### 2.2. Readout Circuit Architecture

**Figure 4:**
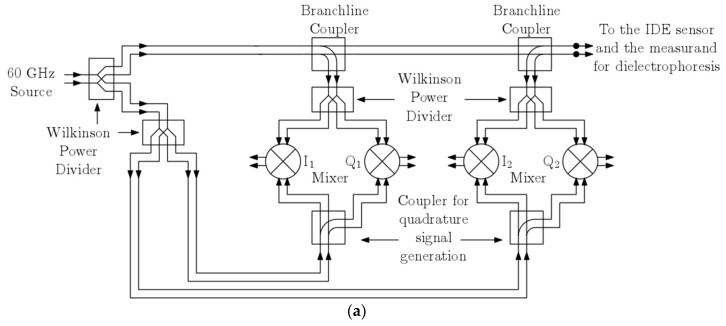

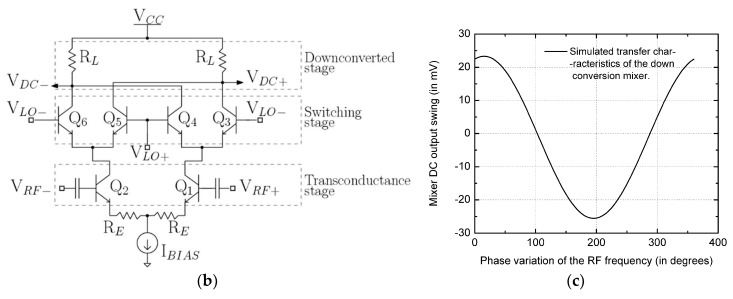
(**a**) High frequency 60 GHz integrated homodyne reflectometer; (**b**) The Gilbert-cell mixer; (**c**) Sample transfer function characteristics of the mixer.

The prospective architecture of the silicon integrated readout circuit is briefly described in this section. Deployment of a high frequency 60 GHz read-out circuit presented in [26] is projected as it allows for miniaturization of sophisticated read-out architectures. The readout circuit is chosen to operate at 60 GHz for it provides a good trade-off between miniaturization and power consumption. A low-frequency implementation of the reflectometer will require a large die area. A homodyne reflectometric scheme to sensor read-out will be accomplished, as shown in Figure 4a, in that the local oscillator (LO) and RF input to the mixers are the same frequency. The major design blocks include a millimeter wave oscillator source, down active conversion mixers, and high-frequency wideband directive elements realized as passives. The two major passives are distributed branch line couplers and Wilkinson power dividers. These have been designed on the topmost metal layer of the technology stack to minimize the transmission losses. The two critical power splitters deployed and indicated as branch line couplers split the input source power for source and reflection characterization. The architecture in Figure 4a comprises two major channels viz. source characterization I/Q mixer channel (I1-Q1) and the reflection characterization I/Q mixer channel (I2-Q2). The source oscillator will excite the load, which is an interdigitated capacitor sensor. Based on the dielectrophoretic action, the immobilized particles will cause a change in impedance of the sensor, which will, in turn, produces a corresponding change in the reflection parameters captured by the reflection channel. Thus, a varying dielectric sample on this sensor will induce a corresponding change in the magnitude and phase of the reflected signal which can be deduced from the reflection channel dc output voltages. The mixers form the major constituent in this architecture and are described here briefly. An active double balanced Gilbert cell mixer is used, and its schematic diagram is shown in Figure 4b. The transistors Q1–Q2 constitutes the input transconductance stage of the mixer. The cascode stage constituting two pairs of transistors, i.e., Q3–Q4 and Q5–Q6, forms the commutating switch. Since the architecture is a homodyne reflectometer, the outputs are dc voltage and are read across the resistive load RL of the mixer. For a possible operating condition of a LO drive of −2.7 dBm and an incident RF power of −19 dBm, the transfer characteristics for an RF phase sweep in the simulation is shown in Figure 4c. 

The integration of the sensor and the high-frequency readout will be accomplished through a dual excitation scheme, which is beyond the scope of this discussion. The measurement noise floor of this architecture is merely governed by the 1/f-noise of the mixers as it is a homodyne reflectometer architecture. Since the heterojunction bipolar transistors (HBTs) have a lower 1/f-noise corner frequency than CMOS, the deployed mixer-based architecture will offer superior noise performance. Additionally, the load resistance of the mixer also contributes to the total output noise. The observed total output noise in the simulation is found to be 2.2 mV_rms_. This governs the minimum cell concentration, which can be reliably handled with the reflectometric readout circuit.

### 2.3. Silicon Microfluidic Integration

**Figure 5:**
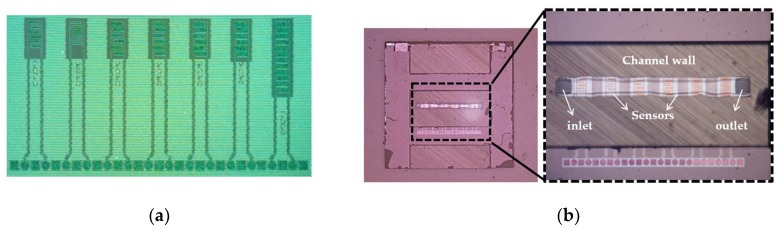
(**a**) Processed IDEs (sensors) structures b; (**b**) Complementary metal-oxide-semiconductor (CMOS) integrated microfluidic lab-on-a-chip device with size of 5 × 5 mm^2^.

The CMOS IDE, as shown in Figure 5a, is combined with a microfluidic channel on a single chip (see Figure 5b). This CMOS integrated microfluidic technology platform improves the functionality and precision by combining the fluidic solution and electrical components. The major step in the integration of the microfluidic channel into the CMOS process is the lines similar to [27] and is briefly repeated. The technology approach integrates CMOS circuitry with microfluidics, based on the 3-wafer-stack technique in a CMOS compatible manner. 

State-of-the-art of our integrated silicon microfluidic CMOS lab-on-a-chip is based on the compatibility of IHP’s CMOS as well as hetero-integration technology of the microfluidic device. The compatibility of CMOS technology can be explained in two aspects. First, how to choose new materials to prevent contamination issues. Second, the CMOS line packaging tool, a so-called pilot package line. The fabrication of CMOS integrated microfluidics is performed without contamination problems of the CMOS process line. The hetero-integrated CMOS technology enables the fabrication of sensors, microfluidic channel, and circuitry on a single chip. This Si-based microfluidic channel is a reliable replacement for the polymer-based microfluidic channel that lacks from the integration of the sensor and circuitry on the same chip. The integration of CMOS circuitry, silicon channels, and glass wafers is done by 200 mm wafer bonding technologies. First, the fabricated the CMOS wafer and the channel wafer are bonded by plasma activated oxide-oxide fusion bonding, followed by adhesive bonding of a glass wafer.

**Figure 6:**
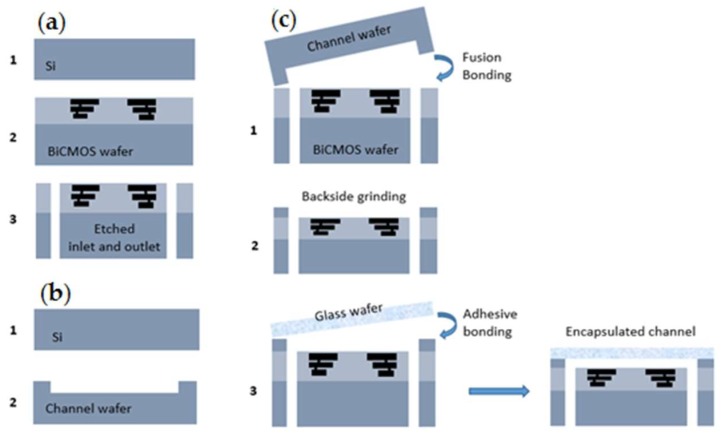
Packaging process of microfluidic lab-on-a-chip: (**a**) CMOS fabrication; (**b**) Channel formation; (**c**) Bonding process by a three-wafer-stack approach [28].

Figure 6 illustrates the entire process flow for microfluidic devices. The wafer with the CMOS devices, including the BEOL sensors is processed on 200 mm Si substrates, Figure 6a (1,2). In the next step, the inlet and outlet ports are opened from the back side of the wafer by localized backside etching, Figure 6a (3). Another bare silicon substrate is used to structure the channel by etching, Figure 6b. The Si-based microfluidic channel and the CMOS wafer are bonded together from their front sides using a low-temperature fusion bonding at 300 °C, Figure 6c (1). After this step, the height of the channel is adjusted by grinding the backside of the microfluidic channel, Figure 6c (2). Finally, for encapsulating of the microfluidic channel, a glass wafer is adhesively bonded to the back side of the channel wafer at 200 °C, Figure 6c (3) [28]. The transparency of the top layer enables the simultaneous optical investigation and electrical sensing measurements. Figure 5b illustrates the fabricated 5 × 5 mm^2^ diced chip with the inlet and outlet and the sensors which are embedded in the channel. One of the noteworthy advantages of using this microfluidic integration technique is the separate interfaces for microfluidics and the electrical connections, enabling highly miniaturized LOC systems. State-of-the-art of this BiCMOS technology offers the opportunity of immobilizing, sensing, and detecting the particles on the same chip. The operational simplicity and low voltage requirement of the DEP technique, as well as the small volume of sample for testing, facilitate the portability of the LOC devices even out of the non-laboratory conditions.

## 3. DEP Analyses and Optimization

**Figure 7:**
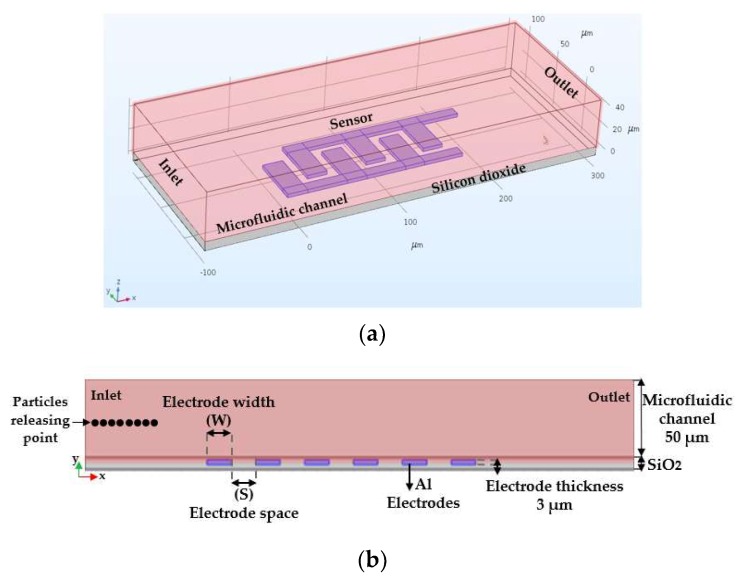
(**a**) 3D-geometry and; (**b**) 2D-geometry of the model device used to study the dielectrophoretic forces on particle tracing.

Based on the COMSOL Multiphysics^®^ tool, 2D and 3D models are developed. To simulate the dielectrophoretic immobilization of particles suspended in an aqueous solution, the fluid flow is considered through a microfluidic channel with a single inlet and a single outlet. Arrays of IDEs are utilized to create a non-uniform electric field that impacts the trajectory of particles due to dielectrophoretic forces. The DEP force was optimized with respect to different geometrical parameters of electrodes, size of the particle, and flow velocity. Figure 7a illustrates the device design in 3D geometry, while the simulation studies were all done in 2D to reduce the computational time, as illustrated in Figure 7b. In this simulation, different geometrical parameters of electrodes, such as width and spacing between adjacent fingers of IDE, was studied. The electric current (ec) module and particle tracing for fluid flow (fpt) in conjugation with drag and dielectrophoretic forces modules were used as the physics interfaces [29].

Different equations were used to simulate the flow path of the particles suspended in the fluidic medium and subsequently trapping them on the electrodes by DEP. The creeping flow (spf) module was used to model the fluid flow through the channel. Fluid velocity within the channel was determined based on the Navier–Stokes Equation shown in (3).

(3) $$0=\nabla \cdot \left[-pl+\mu \left(\nabla u+\left(\nabla u\right)T\right)-\frac{2}{3}\mu \left(\nabla \cdot u\right)l\right]+F\;\nabla \cdot \left(\rho u\right)=0.$$

Here, *p* is the pressure, *u* is the velocity vector, *µ* is the dynamic viscosity, and *F* is the volume force vector that is acting on the fluid. When a particle is suspended in the fluid, it is affected by several forces. One of these forces, is called the drag force, and is caused by the fluid flow and has the same direction as the flow. The drag force is calculated based on Stokes drags law, which is also applicable for creeping flows (Re_r_ « 1), as shown in Equations (4) and (5).

(4) $$F_{Drag}=\left(\frac{1}{T_{p}}\right)\cdot m_{p}\left(u-v\right),$$

(5) $$\tau_{p}=\rho_{p}\cdot {\frac{d_{p}}{18\mu }}^{2},$$

where $\tau_{p}$ is the velocity response time of particles, $m_{p}$ is the particle mass, ν velocity of the particles, *u* fluid velocity, μ fluid viscosity, $\rho_{p}$ particle density, and $d_{p}$ particle diameter. Furthermore, to attract and trap the particles, the dielectrophoretic force (*F_DEP_*) is required, which is given by Equation (6). The particles were subjected to a non-uniform AC electric field.

(6) $$F_{DEP}=2\pi r_{p}{}^{3}{ɛ}_{f}real\left(CM\right)\nabla |E|,$$

(7) $$CM=\frac{\left({ɛ}_{p}{}^{*}-{ɛ}_{f}{}^{*}\right)}{\left({ɛ}_{p}{}^{*}+2{ɛ}_{f}{}^{*}\right)},$$

(8) $$\varepsilon_{f}^{*}={ɛ}_{r}–\left(\frac{i\sigma }{\omega }\right).$$

Here, $r_{p}$ is the particle radius, $\varepsilon_{f}$ is the relative permittivity of the medium, $\varepsilon_{f}^{*}$ and $\varepsilon_{p}^{*}$ are the complex permittivities of the fluid and particles, respectively. *E* is the root-mean-square of electric field strength. Permittivity is a complex quantity which is a function of the electric field’s angular frequency (*ω*) and conductivity (*σ*). Based on the electric potential (*Ʋ*) which is applied to the electrodes, the electric field E, is simulated by the ec module and is calculated based on Equation (9).

(9) E=∇υ.

The fpt module, together with the particle tracing, contains the equations governing the motion and trajectory of particles in the fluid under the influence of a DEP force and drag force. By ignoring the gravity force, buoyancy and Brownian motion forces, the relevant equation can be written, as shown in (10).

(10) $$m_{p}\frac{dv}{dt}=F_{DEP}-F_{Drag}=F_{T},$$

where, $m_{p}$ is the mass of particles, v is the velocity of the fluid, and *F_T_* is the total force acting on the particle.

An electrically insulated boundary condition is applied to the fluidic boundary, which is active on all exterior boundaries of the model. Negative and positive electric potentials were imposed on the electrodes at the bottom of the microfluidic channel. The electric potential of zero Volt was then applied to the electrodes as a further initial condition. The wall conditions were imposed without slip. No slip boundary condition (*u* = 0) was used to model solid walls and bounce for modeling the tracing of microscopic particles in the fluid. The position of the inlet and the outlet were perpendicular to the device structure. The boundary condition for the fluid flow at the inlet was set as velocity and at the outlet was set as pressure. At the inlet, the initial fluid flow velocity ($v_{0}$) was set to 50 µm/s. This value was then varied to study its impact on the immobilization of particles. To establish a fluid pressure gradient in the channel, an initial fluid velocity was applied at the inlet and the outlet was kept to zero fluid velocity with suppressing backflow of the medium. For tracing the particles, disappearing or freezing wall conditions were selected at the outlet. The disappearing mode was used when the particles leave the channel in case of deviation from the electrodes. The freezing wall condition was used to study the velocity and the position of particles, where particles could stay frozen at the point where they leave the channel outlet boundary.

**Figure 8:**
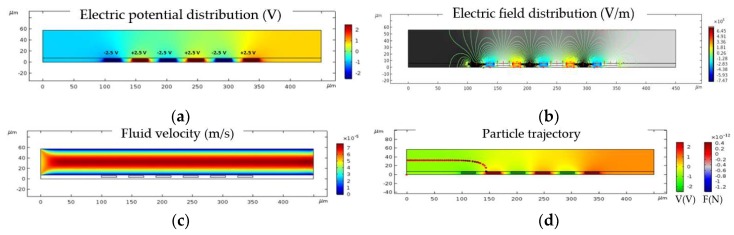
Simulation analysis steps for 3.5 µm diameter size particles: (**a**) Electric potential distribution in the microfluidic channel at *Ʋ* = 5 V at a fixed frequency of 10 MHz; (**b**) Electric field distribution contour plot in the microfluidic channel; (**c**) Velocity field across the microfluidic channel; (**d**) Particle trajectories with respect to the applied electric potential and the force acting on the particles leading to the immobilization of the particles at the surface of electrodes.

The simulation analysis was done in three main steps. The electric current module, which simulates the electrical potential field, was applied to the electrode to create a non-uniform electric field. A stationary analysis was performed to simulate the flow velocity through the channel, as illustrated. Particle tracing module was used to model dielectrophoretic force through the channel. Figure 8 describes an example of the simulation analysis steps for 3.5 µm diameter sized particles. As shown in Figure 8a, negative and positive voltage peak values of 2.5 V and −2.5 V were applied to the electrodes. Therefore, the total applied electric potential was 5 V peak to peak voltage (*Ʋ* = 5 V = V_p-p_). Figure 8b shows the electric field contour plot. Figure 8c illustrates the constant fluid flow velocity in the middle part of the channel. The pressure was almost constant in the channel. For particle tracing a time-dependent solution was performed using the values obtained from the frequency domain analysis, as shown in Figure 8d.

Since the objective of this study is to immobilize the particles on the electrodes, simulations were performed systematically to optimize the electrode geometry and to find suitable electric field frequency ranges and fluid velocities to attract different size particles. 

The particle sizes used for these simulation studies were in micron and submicron-diameter ranges (10, 3.5, 0.5). These particles were used as models and assumed as homogeneous spheres and non-biological. To keep the first model simple and reduce the complex parameters, it was assumed that these particles were diluted in water. Therefore, the osmotic pressure effect can be neglected for these simulations. Table 2, represents the properties of the particles for the simulations. 

### 3.1. Impact of Frequency on DEP

**Figure 9:**
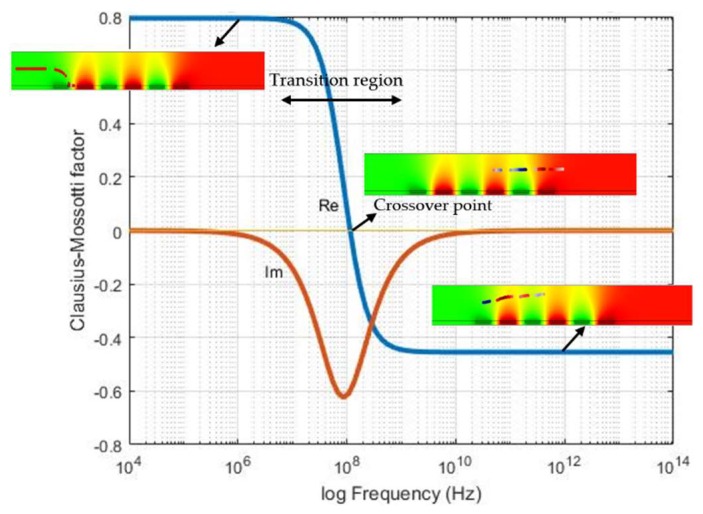
Clausius Mosotti (CM) factor for 10 µm diameter-sized particle as a function of frequency.

The choice of the operating frequency, which leads to immobilization of the particles, was based on the Clausius Mossotti (CM) factor. CM is a frequency dependent parameter which results in negative and positive DEP. Changing the frequency stimulate the crossover frequency between positive and negative DEP. The value of the real part is varied between +1 and −0.5. When the real part of the CM factor is positive, positive DEP forces act on the particle, which results in the attraction of them to the electric field intensity maxima, i.e., electrode edges. In the opposite case (real [CM] < 0), particles are repelled from high electric field intensity regions and consequently deviated from the electrode. To find a suitable frequency range, the CM factors of the particles were calculated by using MATLAB^®^. Figure 9 illustrates the CM factor for 10 µm diameter size fluorescent particles. Particle properties are shown in Table 1. At positive values of the Clausius Mossotti factor (real [CM] > 1), particles are trapped on the electrodes. The positive DEP is caused by the positive values of CM. At negative real values [CM] < 0, negative force is acting on the particles. This leads to the deviation of particles from the electrodes. Therefore, for reliable DEP immobilization, frequencies below 10 MHz must be chosen. 

### 3.2. Impact of Voltage Variation and Particle Size

**Figure 10:**
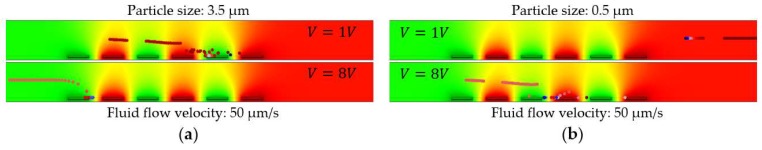
Impact of voltage variation on the DEP force for particles at constant fluid flow velocity (50 µm/s), geometrical parameters of IDEs (electrode width of, 30 µm, and spacing of, 20 µm) and DEP operating frequency (1 MHz). (**a**) Diameter size of 3.5 µm; (**b**) 0.5 µm.

The simulated results of the improved particle tracing for 3.5 and 0.5 µm diameter-size particles are shown in Figure 10. The immobilization of particles was positively correlated with the voltage. For the same IDE structure, the immobilization of 3.5 µm size particles started already at 1 V, Figure 10a, while the immobilization of 0.5 µm particles occurred at 8 V, shown in Figure 10b. 

The dielectrophoretic force which is subjected to the particles by the electric field is positively correlated with the electric potential. In other words, for the trapping of smaller particles on electrodes, higher voltages are required to compensate the mechanical force and overcome the drag force from fluid flow.

### 3.3. Impact of Fluid Flow Velocity

**Figure 11:**
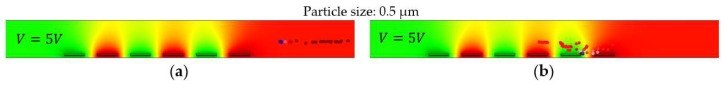
Influence of fluid flow velocity on tracing of the 0.5 µm particles through the microfluidic channel at a constant voltage of 5 V and operating frequency of 1 MHz. At flow rates of: (**a**) 50 µm/s; and (**b**) 30 µm/s.

The maximum operating voltage of IHP CMOS technology is constrained to 5 volts. To immobilize the submicron particle, the voltage was kept at CMOS compatible ranges, and the fluid flow velocity was varied. The fluid velocity is inversely impacted by the immobilization of small particles. Figure 11 shows a representative simulation result for reducing the fluid velocity at a constant voltage of 5 V. The fluid flow velocity of 50 µm/s was set as the reference. It can be seen that reducing the fluid velocity to 30 µm/s improved the attraction of particles. 

### 3.4. Impact of IDE’s Geometry on DEP Immobilization Probability

**Figure 12:**
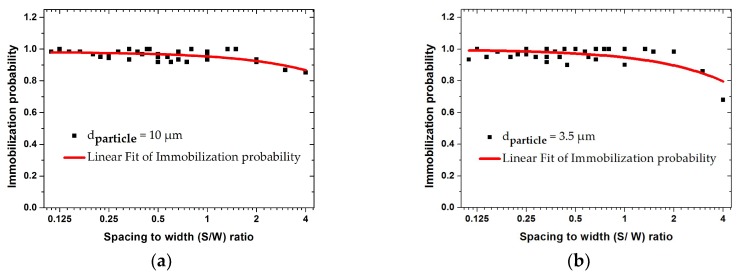
Finite element modeling (FEM) results for the immobilization probability with different ratios of the IDE’s spacing and width ($\frac{S}{W}$) for 10 and 3.5 µm-sized particles at 1 MHz and fluid velocity of 50 µm/s: (**a**) 10 µm; (**b**) 3.5 µm particle.

In the following simulation step, the voltage, flow velocity, and the frequency were kept constant. The impact of the geometrical dimensions of the IDE on the trajectory of particles is studied. The immobilization probability values of each IDE’s geometrical design can be calculated by COMSOL^®^ applications. Based on these analyses, the immobilization probability is defined as the ratio of the number of immobilized particles to the total number of particles suspended in the medium. The influence of the fringing field effect with different ratios of the electrode width and spacing ($\frac{S}{W}$) versus immobilization probability is shown in Figure 12. 

**Figure 13:**
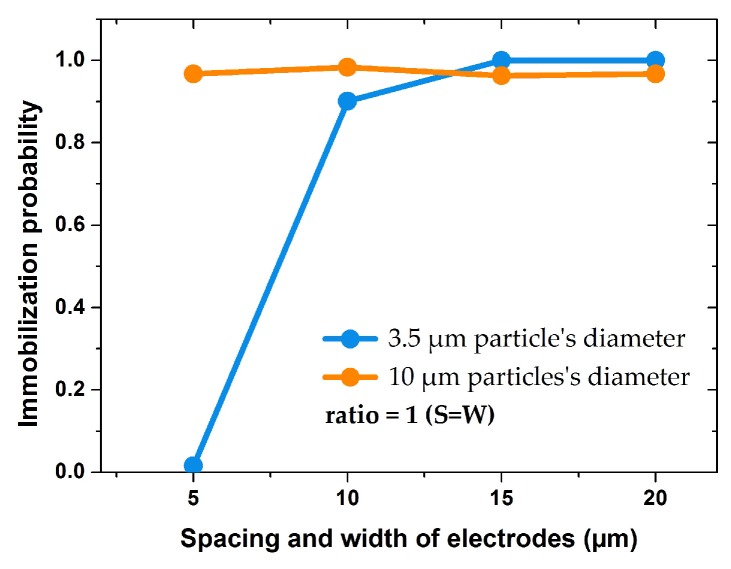
Impact of IDE’s width in symmetric structures (S=W) on immobilization probability of 10 and 3.5 µm particles under the same voltage and fluid velocity.

**Figure 14:**
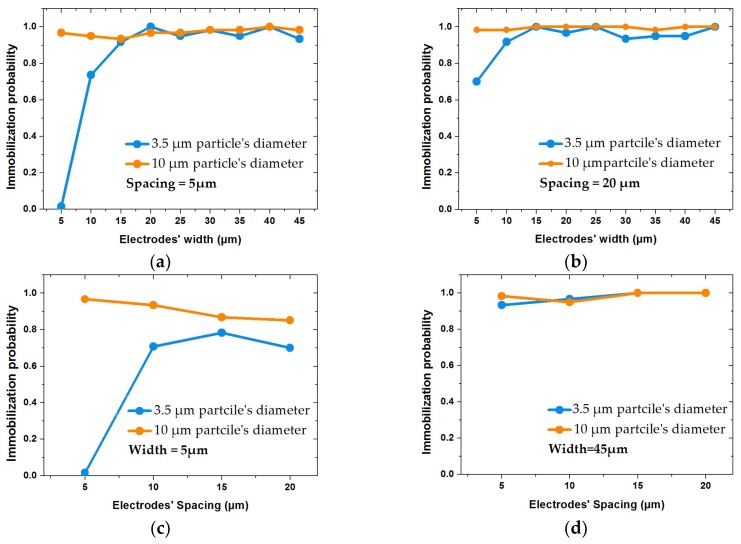
Impact of IDE’s geometrical parameters on immobilization probability (IP) of particles with diameters of 10 and 3.5 µm, keeping voltages and fluid velocities constant: (**a**,**b**) illustrate IP variations with respect to different widths at fixed spacings of 5 and 20 µm, respectively; (**c**,**d**) show the impact of spacing size on IP at fixed widths of 5 and 45 µm, respectively.

The width varied between 5 and 45 µm, and the spacing varied between 5 and 20 µm. These graphs illustrate the immobilization probability (IP) of 10 and 3.5 µm size particles. For both particle sizes, IP was larger than 0.9 for ratios below 2. At higher ratios, IP values decreased for both particle sizes. This reduction becomes significant for smaller particles at a ratio of 4. The IDE design was optimized when the ratio of spacing to width is smaller than 2. In the following section, this trend is explicitly discussed and illustrated in Figure 13 and Figure 14. 

In the following, the IDEs are assigned into two groups of uniform and non-uniform geometries. Uniform IDEs refers to geometries with equal sizes of width and spacing. Geometries with an unequal size of width and spacing are referred to as non-uniform IDEs. Figure 13 shows the IP values for uniform IDEs. 

Using the uniform electrode geometries, the immobilization probability for larger particles (10 µm) was almost 100% in all cases. For smaller particles (3.5 µm) IP increased when the electrodes width and spacing were larger than 10 µm. For IDEs with spacing and width of 5 µm, IP was zero for small particle, whereas for bigger particle it was almost 1. Therefore, it can be concluded that the uniform IDEs design is optimized when spacing and width are larger than 10 µm. 

Figure 14 illustrates the impact of non-uniform IDE structures on immobilization probability of particles with sizes of 10 µm and 3.5 µm. Figure 14a,b represent the IP at a constant spacing of 5 and 20 µm. Figure 14c,d illustrate the IP at constant widths (5 and 45 µm) with respect to spacing. The variation of spacing and width has less impact on IP of 10 µm size particles. However, for small particle, 3.5 µm, the results were different. Keeping the spacing constant at 5 µm, IP decreased drastically, as shown in Figure 14a. However, this effect inversely impacts the IP with respect to spacings, which were comparatively much larger than the width (ratios over 2), as shown in Figure 14c. It can be concluded that the impact of the geometrical parameters is dominant if small particles have to be immobilized. Increasing spacing positively impacts the trapping of small particles for non-uniform IDEs ratios ($\frac{S}{W}$) below 2. In other words, the fringing field effect positively affects the probability to immobilize smaller particles with increasing width. 

Based on the results, the optimum geometrical parameters were selected and applied for the simulation of the submicron particle (0.5 µm). These structures’ parameter with their respective spacing to width ratios are shown in Table 3. For these simulations, the applied voltage was kept constant at CMOS compatible range (5 V), and the fluid flow velocity reduced to 30 µm/s. 

**Figure 15:**
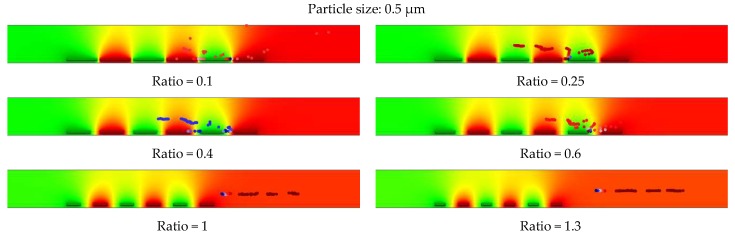
Impact of the optimized parameters (geometrical parameters and fluid flow velocity) on immobilization of submicron particles (0.5) µm at constant voltages of 5 V and fluid flow velocity of 30 µm/s.

The tracing behaviors of 0.5 µm particles for selected geometries are shown in Figure 15. The same tracing trends as micron-sized particles were observed for submicron-sized particles at reduced flow rates for structures with ratios below 1. The decreased spacing to width ratios improved the tracing and immobilization of submicron size particles. Using higher ratios, particles were repelled from the electrodes and left the microfluidic channel.

Therefore, it was observed that designing a better performance IDE is highly influenced by the optimum ratio of the electrode’s spacing to width. Furthermore, the flow rate of the medium inside the microfluidic channel impacts the particles’ movement behavior inside the electric field. These are important factors to consider for the immobilization of submicron and micron size particles. The optimized IDE parameters for immobilization of submicron particles are related to IDE geometries with ratios below one. However, for immobilization of micron size particles, the IDE is optimized for ratios below two.

### 3.5. Impact of Temperature on DEP Process

**Figure 16:**
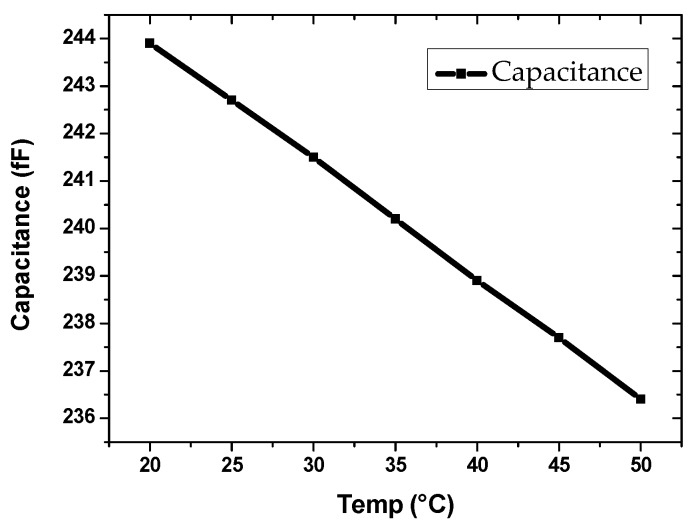
IDE’s capacitance vs. water dielectric permittivity [30] at 1MHz excitation frequency, observed from room temperature up to 50 °C.

An insightful study of temperature effects on IDE parameters in the vicinity of room temperature is investigated though electromagnetic momentum simulation studies. Water was the liquid carrier of the particles, and thus, the impact of the prospective chip self-heating on the dielectric permittivity change is illustrated. Figure 16 shows the capacitance of the IDE due to change in water dielectric from room temperature up to 50 °C. The frequency of observation remained 1 MHz, and the water dielectric layer thickness considered here was 50 μm corresponding to the height of the microfluidic channel. The change in capacitance due to a temperature of up to 50 °C was found to be only up to 3.07%. The temperature effects due to self-heating of the chip may be a pronounced issue for very long duration measurements only. However, the use of active circuit components is judicious to be used sparingly in the read-out circuit for particle detection. For instance, the source oscillator was an external signal source so that one of the power-consuming blocks is eliminated from the chip. 

The total power consumption was merely 64.3 mW and is contributed by the mixers. Additionally, the chip can be operated in a short duty cycle when the measurement of the change in capacitance is to be conducted. This ensures reliable operation which is minimally impacted by the chip-self heating and more so when the low power consumption readout circuit is placed away on the chip, relative to the sensors.

## 4. Conclusions

The viability of the CMOS embedded microfluidic device for dielectrophoretic immobilization of particles was investigated by finite element modeling (FEM) simulations. This study proposed a sensing platform to enable the immobilization of particles with micron and submicron sizes constrained by technological advances, such as silicon-based microfluidics and millimeter wave readout circuit. IDE’s geometrical parameters were characterized and optimized based on essential external parameters, such as voltage, frequency, and velocity of the fluid through the microfluidic channel. By increasing the applied voltage trapping of smaller particles on electrodes is enhanced. The change of frequency does not influence the DEP force for improving the immobilization of particles but rather influences the crossover frequency between negative and positive DEP. Reducing the fluid flow velocity forces the immobilization of submicron particles. By keeping the voltage constant at CMOS compatible range, the impact of IDE’s geometrical parameters on the tracing and immobilization of the particles was investigated. The ratio between the electrodes’ spacing and the width impacts the IDE’s performance. According to simulations, the width of the IDE’s finger has to be increased, whereas the spacing has to be reduced. The simulation and design of planar IDEs were evaluated to maximize the immobilization probability of submicron sized particles. The IDE design for the immobilization of micron and submicron size particles with optimum geometrical parameters of IDE and the fluid flow rate was demonstrated. Furthermore, a study on the impact of temperature (also due to chip self-heating) in IDE parameters was investigated though electromagnetic momentum simulations. It was observed that the change in impedance of IDE is only up to 3.07℅ from room temperature up to 50 °C which is less likely to affect the short term measurements. Lastly, the presented optimized IDEs can be combined with various readout architectures. The 60 GHz high-frequency reflectometer based architecture was chosen to enable higher miniaturization of a sophisticated sensing platform, and its homodyne architecture will enable a dc-in and dc-out based impedance spectroscopy.

## Figures and Tables

**Table 1:** Comparison of complementary metal-oxide-semiconductor (CMOS) integrated lab-on-chip systems.

Ref.	Particle	Aim	Particle Manipulation Platform	Particle Detecting	Readout Circuitry
[7]	Auto fluorescent protein R-phycoerythrin (RPE)/fluorescently labeled IgG Antibody	immobilization	more than 100 K cylindrical sub-microelectrodes	monitoring fluorescence intensity	potential for on-chip
[16]	Living cells 20–30 µm	immobilization	102,400 actuation electrodes	optical sensor	on-chip
[17]	Micro-nano particles 500 nm–10 µm	separation	L-shaped electrode	IDE ^1^	off-chip
[18]	Yeast cells	immobilization	octa-pole electrode	trans-impedance amplifier	off-chip
[19]	Fat and calcium in the blood	plaque characterization in arteries	-	IDE ^1^	potential for on-chip
[20]	Yeast cells	detecting cell concentration	-	IDE ^1^	on-chip
[21]	A mixture of glycerol/water and glycerol/alcohol	detecting the variation of fluid’s relative viscosity	-	IDE ^1^	on-chip
[22]	Yeast cells	particle counting/single particle sensing	-	IDE ^1^	on-chip

^1^ Interdigitated electrodes.

**Table 2:** Particle properties.

MUT ^1^	Particle Diameter (µm)	Density (kg/m^3^)	Permittivity	Conductivity (S/m)
Particle 1 (platelet)	3.5	1000	50	25
Particle 2	0.5	1000	50	25
Particle 3 (fluorescent)	10	1050	5	0.69

^1^ Material Under Test.

**Table 3:** Selected IDE geometries which are compatible with CMOS operating range (5 V).

IDE Structure	Structure 1	Structure 2	Structure 3	Structure 4	Structure 5	Structure 6
Electrode width (W)	45 (µm)	40 (µm)	35 (µm)	30 (µm)	20 (µm)	15 (µm)
Electrode spacing (S)	5 (µm)	10 (µm)	15 (µm)	20 (µm)	20 (µm)	20 (µm)
Ratio = S/W	0.1	0.25	0.4	0.6	1	1.3
